# Molecular subtypes based on cuproptosis regulators and immune infiltration in kidney renal clear cell carcinoma

**DOI:** 10.3389/fgene.2022.983445

**Published:** 2022-10-21

**Authors:** Aibin Liu, Yanyan Li, Lin Shen, Na Li, Yajie Zhao, Liangfang Shen, Zhanzhan Li

**Affiliations:** ^1^ Department of Geriatrics, Xiangya Hospital, Central South University, Changsha, China; ^2^ Department of Nursing, Xiangya Hospital, Central South University, Changsha, China; ^3^ Department of Oncology, Xiangya Hospital, Central South University, Changsha, China; ^4^ Department of Nuclear Medicine, Xiangya Hospital, Central South University, Changsha, China; ^5^ National Clinical Research Center for Geriatric Disorders, Xiangya Hospital, Central South University, Changsha, China

**Keywords:** cuproptosis, KIRC, immune infiltration, immunotherapy, molecular subtypes

## Abstract

Copper toxicity involves the destruction of mitochondrial metabolic enzymes, triggering an unusual mechanism of cell death called cuproptosis, which proposes a novel approach using copper toxicity to treat cancer. However, the biological function of cuproptosis has not been fully elucidated in kidney renal clear cell carcinoma (KIRC). Using the expression profile of 13 cuproptosis regulators, we first identified two molecular subtypes related to cuproptosis defined as “hot tumor” and “cold tumor”, having different levels of biological function, clinical prognosis, and immune cell infiltration. We obtained three gene clusters using the differentially expressed genes between the two cuproptosis-related subtypes, which were associated with different molecular activities and clinical characteristics. Next, we developed and validated a cuproptosis prognostic model that included two genes (FDX1 and DBT). The calculated risk score could divide patients into high- and low-risk groups. The high-risk group had a poorer prognosis, lower level of immune infiltration, higher frequency of gene alterations, and greater levels of FDX1 methylation and limited DBT methylation. The risk score was also an independent predictive factor for overall survival in KIRC. The established nomogram calculating the risk score achieved a high predictive ability for the prognosis of individual patients (area under the curve: 0.860). We then identified small molecular inhibitors as potential treatments and analyzed the sensitivity to chemotherapy of the signature genes. Tumor immune dysfunction and exclusion (TIDE) showed that the high-risk group had a higher level of TIDE, exclusion and dysfunction that was lower than the low-risk group, while the microsatellite instability of the high-risk group was significantly lower. The results of two independent immunotherapy datasets indicated that cuproptosis regulators could influence the response and efficacy of immunotherapy in KIRC. Our study provides new insights for individualized and comprehensive therapy of KIRC.

## Introduction

Renal cell carcinoma (RCC) is a common malignant tumor derived from renal tubular epithelial cells (Moch et al., 2016). Globally, more than 350,000 new cases of RCC are diagnosed and 140,000 associated deaths are identified per year, representing approximately 2%–3% of all cancer diagnoses and deaths (Gupta et al., 2008; Sung et al., 2021). Kidney renal clear cell carcinoma (KIRC) is one of the most aggressive subtypes of RCC, comprising 70%–80% of all cases of RCC and shows a male preponderance of 2:1 (Capitanio et al., 2019). The standard treatment for surgically respectable patients with KIRC is partial or radical nephrectomy with curative intention (Meskawi et al., 2012). Active surveillance, ablative therapies, and systematic treatments containing radiation therapy, chemotherapy, targeted therapy, and immunotherapy were chosen for patients with inoperable or metastatic KIRC (El et al., 2012; Pierorazio et al., 2015). Immune responses are closely correlated with clinical performance in KIRC (Considine and Hurwitz, 2019). With the advent of immunotherapy, many immune-related drugs, such as nivolumab and ipilimumab, have been applied to patients with KIRC to improve survival with successful outcomes (Motzer et al., 2019). Despite considerable improvements in diagnosis technologies and treatment methods in the past several decades, the prognosis of KIRC patients remains poor due to metastasis at diagnosis and drug resistance.

A recent study reported a novel form of cell death: cuproptosis, through which copper toxicity induces the destruction of certain mitochondrial metabolic enzymes, triggering an unusual mechanism of cell death. This mechanism could explain the pathology associated with inherited copper overload diseases and suggest new ways to exploit copper toxicity to treat cancer (Tsvetkov et al., 2022). Some studies have reported that cuproptosis is associated with cancer prognoses and immunotherapy sensitivity (Wang et al., 2022a). For instance in pancreatic cancer, a model using three cuproptosis genes was shown to be a good predictor of prognosis. Furthermore, the study found significantly different levels of immune infiltration between different risk groups based on the cuproptosis prognosis model (Xu et al., 2022). A recent study also explored the role of cuproptosis in KIRC (Bian et al., 2022). However, some important issues have not been clearly investigated, such as the role of gene alterations, the definition of molecular subtypes, and the development of a prognostic model based on cuproptosis regulators, and potential response to chemotherapy and immunotherapy. In the present study, we investigated gene alterations and variations in the number of cuproptosis regulators genes in KIRC. Next, we performed a clustering analysis based on differentially expressed cuproptosis regulators and explored the clinical and immune characteristics of these molecular subtypes. We then developed and validated a cuproptosis-related prognostic model by calculating a risk score. The risk score obtained could independently predict the prognosis of the individual patient using a novel nomogram risk assessment model. Finally, we explored the effect of the cuproptosis regulator-based risk score on the potential response to chemotherapy and immunotherapy. Overall, we present a new and different perspective on the functional roles of cuproptosis in KIRC, and provide new information for developing an individualized treatment strategy for KIRC.

## Methods

### Data sources

We obtained the KIRC transcriptome profile (tumor: 539, normal: 72 samples) from The Cancer Genome Atlas (TCGA) (https://portal.gdc.cancer.gov/), and clinical information was also extracted, including age, sex, grade, stage, and tumor node metastasis classification (TNM). Data without follow-up time or outcomes were excluded. The dataset included 531 KIRC patients, which were randomly separated into a training group (*n* = 266) and a validation group (*n* = 265), and complete data with clinical data was 530. The general characteristics of data sources were presented in Table 1. Simple nucleotide variation and copy number variation were also downloaded obtained. All omics data were normalized before the analysis. We obtained 13 cuproptosis regulators from a previous study (Supplementary Table S1) (Cobine and Brady, 2022; Li et al., 2022) and the gene list is provided in Supplementary Table S2. All original data and codes have been submitted to the Editorial Office.

**TABLE 1:** General characteristic of data source of patients.

Category	Number	Percent (%)
Age		
<=60	264	49.8
>60	266	50.2
Gender		
Male	344	64.9
Female	186	35.1
Grade		
G1-2	241	45.5
G3-4	281	53.0
GX	5	0.9
unknown	3	0.6
Stage		
I/II	322	60.8
III/IV	205	38.7
unknown	3	0.6
T		
T1-2	340	64.2
T3-4	190	35.8
N		
N0	239	45.1
N1	16	3.0
NX	275	51.9
M		
M0	420	79.2
M1	78	14.7
MX	30	5.7
unknown	2	0.4

### Molecular clustering

The molecular clustering was completed using the R packages “ConsensusClusterPlus”. This method chooses the optimal number of clusters after 1000 repeated calculations. The clustering result was further confirmed by principal component analysis (PCA) and the t-distributed stochastic neighbor embedding (tSNE) algorithm. We first performed molecular clustering in terms of cuproptosis regulators, and then performed gene clustering using differential gene expression (DGE) analysis between the different cuproptosis clusters. Kaplan-Meier analysis was used to compare the overall survival (OS) curves of different molecular clusters.

Gene set variation analysis (GSVA) was performed using the R package “GSVA”. We first calculated the pathway enrichment score of each sample using the gmt file “c2.cp.kegg.v6.2.symbols.gmt” and performed the DGE analysis using the R package “limma”. The results are presented in the form of a heatmap. *p*-values <0.05 were considered a statistically significant. We estimated the correlations among different genes using Spearman’s methods. Gene ontology (GO) and Kyoto Encyclopedia of Genes and Genomes (KEGG) enrichment analysis were performed using the R “clusterProfiler” packages.

### Development and validation of a cuproptosis prognostic model

The entire KIRC dataset was randomly grouped into training and validation groups (50% for each group). The least absolute shrinkage and selection operator (Lasso) analysis was used to identify the signature genes in the training dataset, and the regression coefficient was obtained from Cox regression. We estimate the cuproptosis score using the following formula: risk score = gene1 expression * regression coefficient-1 +... + geneN expression × regression coefficient-N. Based on the median score value, we divided KIRC patients into high- and low-risk groups. We then conducted a survival analysis for the high- and low-risk groups using the “survminer” package R. PCA analysis was used to show the risk distribution and receiver operating characteristic curves (ROC) were performed to calculate the area under the curve (AUC) for 1-year, 2-year and 3-year OS and to evaluate the predictive capacity of the signature. Using the established prognostic model, we validated the results of all analyses in the validation data set.

### Independence and clinical correlation analysis

To explore the independence of the cuproptosis score in the prognosis of KIRC, we performed a univariate and multivariate Cox regression analysis. The hazard ratio (HR) and the 95% confidence interval (CI) were also calculated. We compared the clinical characteristics among different cuproptosis clusters, gene clusters, and risk groups using the Chi-square test, and compared the cuproptosis score of different cuproptosis and gene clusters using non-parameters test.

To evaluate the prognosis of each patient, we built a nomogram risk assessment model using the risk score and clinical parameters. The calibration plot was used to assess the degree of fitness between observed probability and predicted probability for 1-year, 3-year, and 5-year OS. A ROC was used to assess the predictive capacity of the nomogram score for individual patients.

### Immune infiltration analysis

We calculated immune, stromal, and estimate scores, and tumor purity using the ESTIMATE algorithm (Yu et al., 2021). Furthermore, we investigated the levels of infiltration of 22 immune cell types and the function for each sample. Genes related to immune checkpoints were also evaluated. Differential analysis was performed for cuproptosis groups, gene groups, and risk groups. *p*-values <0.05 were considered statistically significant.

### Chemotherapy sensitivity and immunotherapy

Data were obtained from the Genomics of Drug Sensitivity in Cancer (GDSC). IC50 of 746 small molecules (GDSC: 265) and in 1861 (GDSC: 860) cell lines was collected along with their mRNA expression data from databases. The mRNA expression and drug sensitivity data were merged. Pearson’s correlation analysis was performed to assess the level of correlation between the drug IC50 and mRNA expression (Muttenthaler et al., 2021). *p*-values with adjusted FDR were obtained. A negative correlation indicated that gene expression is suppressed, which suggested sensitivity to that drug and *vice versa*. We also compared IC50 levels between high- and low-risk groups.

Subsequently, we validated the role of the cuproptosis score on prognosis using Imvigor 210 (http://research-pub.gene.com/IMvigor210CoreBiologies/) and the GSE78220 dataset (https://www.ncbi.nlm.nih.gov/geo/query/acc.cgi?acc=GSE78220),which were cohorts of patients with urothelial carcinoma and melanoma treated with an inhibitor of PD-L1 (Hugo et al., 2016; Mariathasan et al., 2018). Follow-up information and expression of the cuproptosis regulator were extracted. Survival analysis was performed to compare the survival curves of different risk scores.

## Results

### Landscape of cuproptosis regulators in kidney renal clear cell carcinoma

**FIGURE 1:**
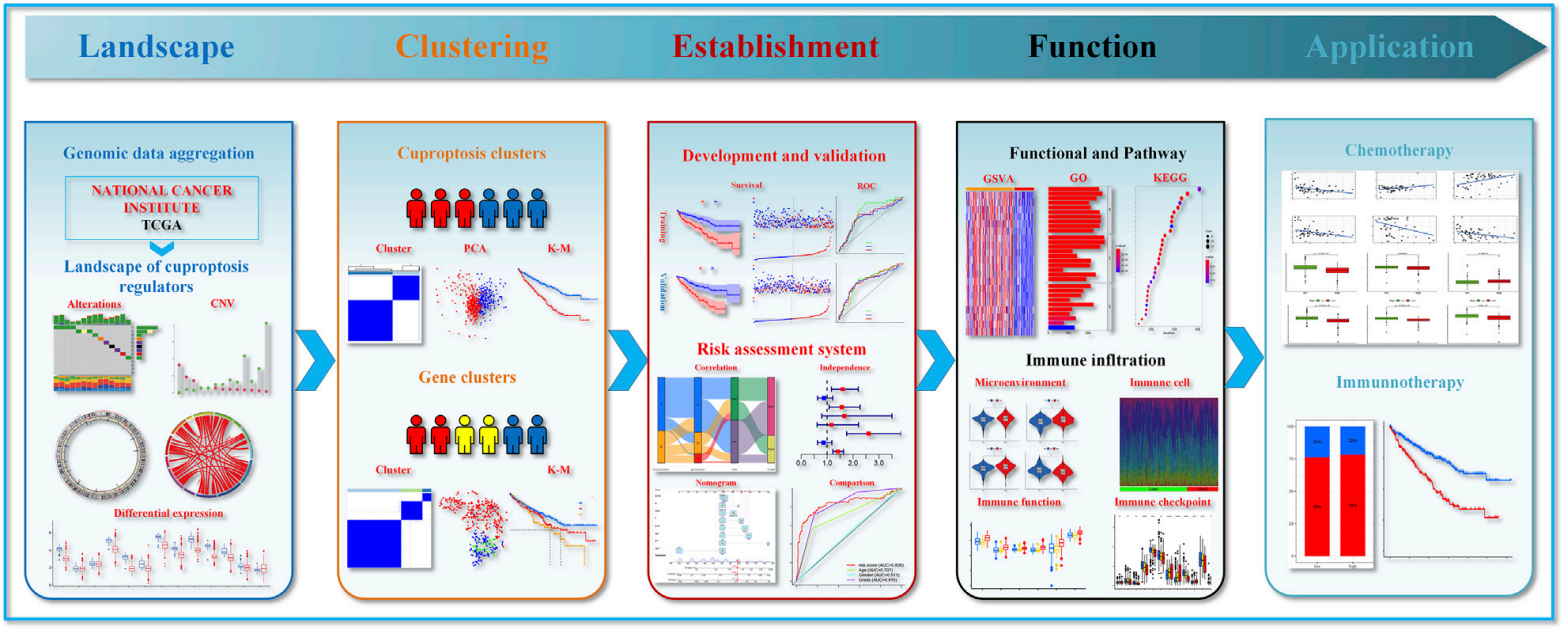
Flow chart showing data processing.

**FIGURE 2:**
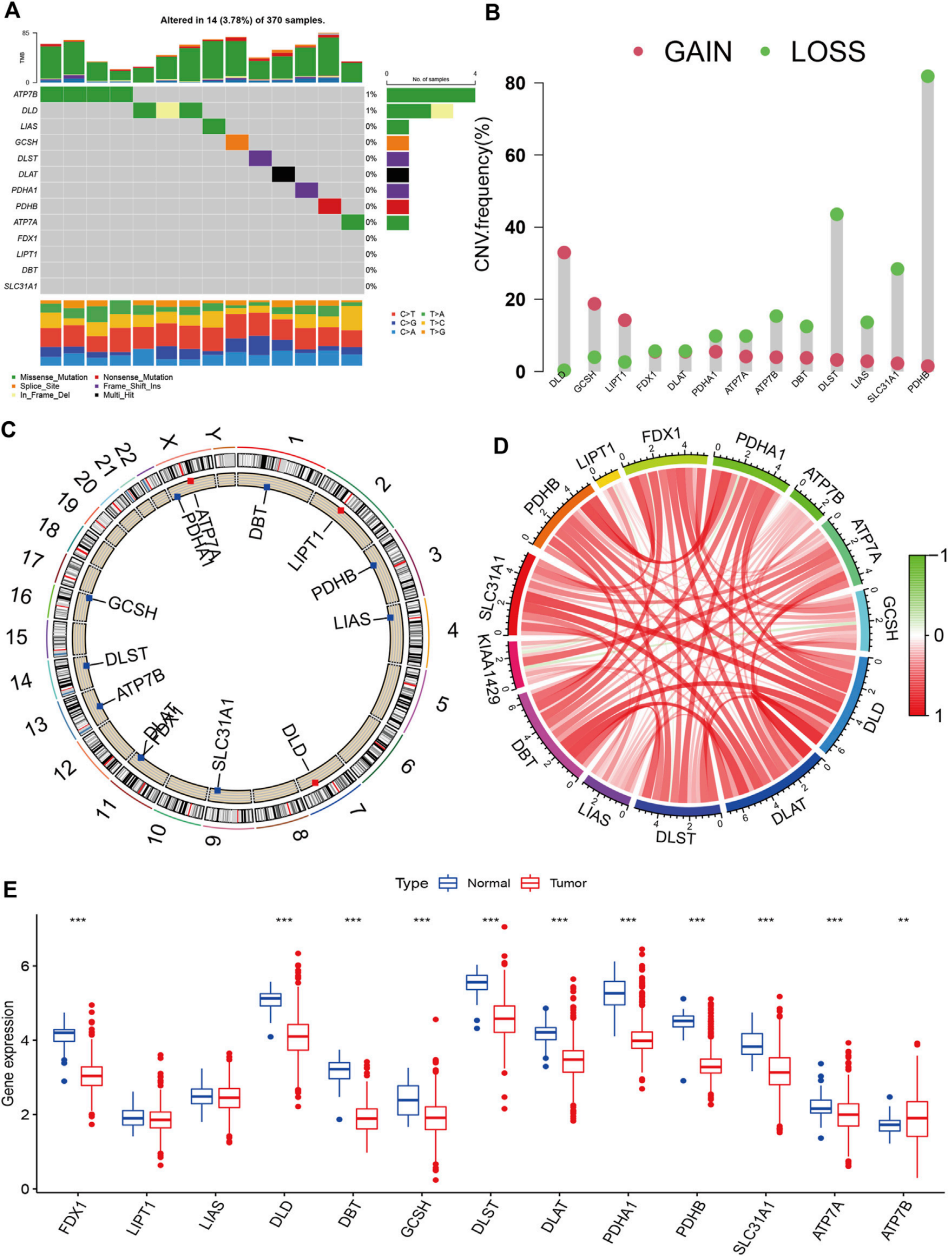
Landscape of cuproptosis regulators in KIRC. **(A)**: Gene alterations of cuproptosis regulators. **(B)**: Copy number variation frequency of cuproptosis regulators. **(C)**: Chromosome ideograms and labelled chromosomes. **(D)**: Correlation circle among cuproptosis regulators. **(E)**: Differentially gene expression analysis of cuproptosis regulators between normal and tumor cells.

Figure 1 presents the entire flow of the data analysis in the study. We first explored the gene alterations of 13 cuproptosis regulators, and identified only two genes (ATP7B and DLD) with a frequency of gene alterations of at least 1% (Figure 2A). Most cuproptosis regulators show a higher loss frequency than a higher gain frequency (Figure 2B). Figure 2C shows the location of these regulators on the chromosome. Among these regulators, most showed positive associations among them, except for GCSH-KIAA1429 (Figure 2D). DGE analyses indicated that most cuproptosis regulators were significantly downregulated in the tumor tissue (Figure 2E).

### Molecular subtypes and characteristics

**FIGURE 3:**
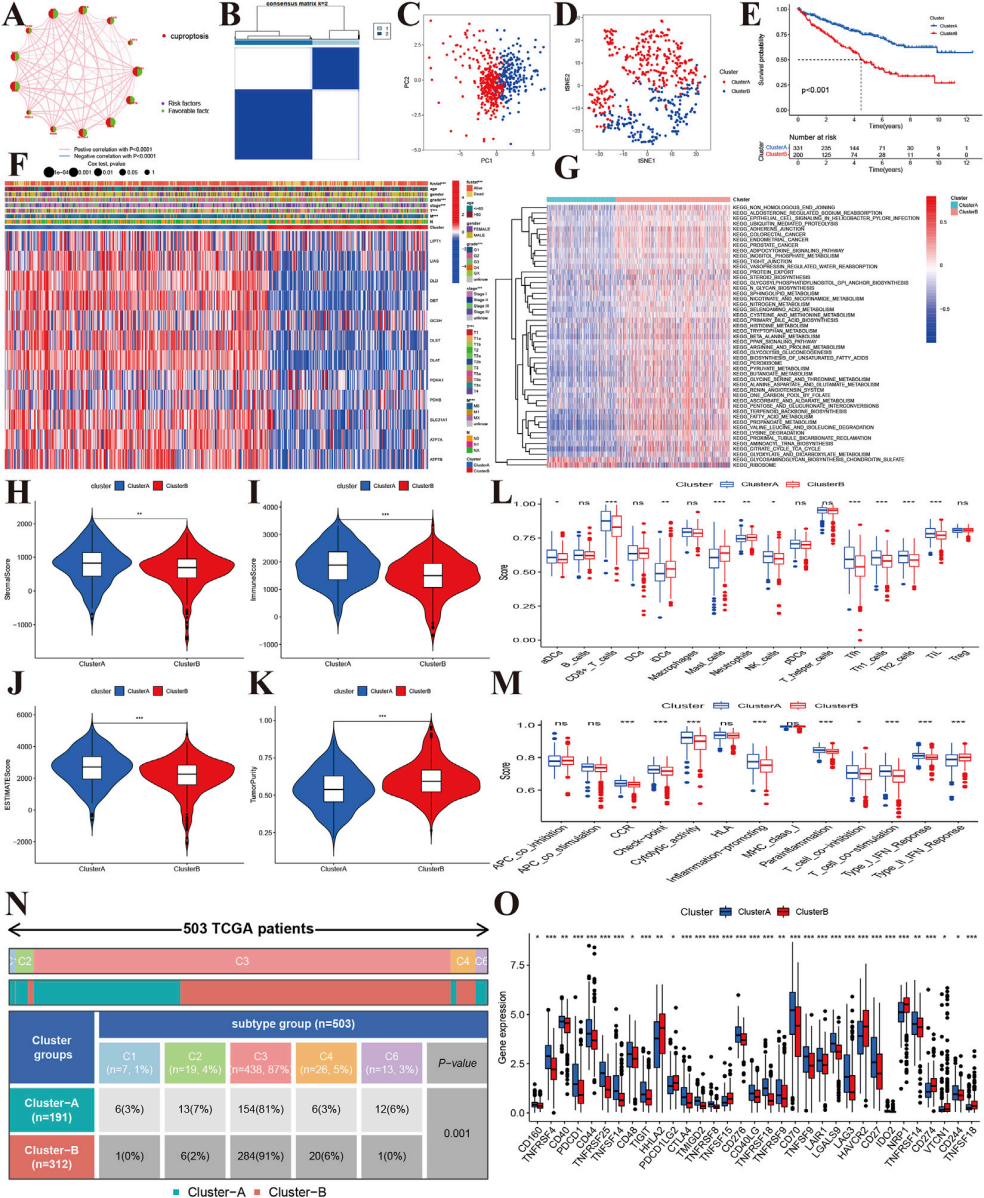
Molecular subtypes based on cuproptosis regulators in KIRC. **(A)**: Correlations between the prognosis of cuproptosis regulators in KIRC. **(B)**: The consensus matrix identified the optimal number of cuproptosis subtypes. **(C,D)**: PCA and tSEN identified two components. **(E)**: Kaplan-Meier survival curves of the two molecular subtypes. **(F)**: Correlations of subtypes with clinical characteristics. **(G)**: GSVA identified differentially expressed signaling pathways. **(H–K)**: Tumor microenvironment and tumor purity levels of different subtypes. **(L,M)**: Immune cells and functions of two cuproptosis subtypes. **(N)**: Distribution of immune subtypes of the two cuproptosis subtypes. **(O)**: Expression levels of immune-related checkpoint genes between the two cuproptosis subtypes.

To explore the molecular subtypes, we first performed survival analyses and identified 12 genes related to prognosis (Figure 3A). Next, we performed consensus clustering and found that KIRC patients could be separated into two cuproptosis clusters (CuproCluster A and CuproCluster B, Figure 3B and Supplementary Table S3). The PCA and tSEN analyses further identified molecular subtypes (Figures 3C,D), and the Kaplan-Meier analysis also indicated that CuproCluster B had poorer OS than CuproCluster A (Figure 3E). We then explored the correlation between clusters and clinical characteristics. CuproCluster B was associated with advanced clinical stage, grade, and T and M classification (Figure 3F), while cuproptosis regulators were highly expressed in CuproCluster B. Finally, we further explored the function and levels of immune infiltration in different molecular clusters. GSVA indicated that most metabolic functions and signaling pathways were significantly and highly enriched in CuproCluster B; epithelial cell signaling, the adipocytokine signaling pathway, and metabolism of glyoxylate and dicarboxylate, glycosaminoglycan biosynthesis, chondroitin sulfate, and ribosome were significantly down-regulated (Figure 3G). Furthermore, we analyzed the tumor microenvironment (TME) and levels of immune infiltration. Our results indicated that the stromal score, immune score, and estimate score were significantly decreased in CuproCluster B, while tumor purity was higher in CuproCluster B than in CuproCluster A (Figures 3H–K). Immune cells including aDC, CD8^+^ T cells, NK cells, Tfh, Th1 cells, The2 cells, and TIL were downregulated in CuproCluster B (Figure 3L). Immune functions (CCR, checkpoint, cytolytic activity, inflammation-promoting, para-inflammatory, co-inhibition of T cells, co-stimulation of T cells, response to Type I IFN) were also significantly decreased in CuproCluster B (Figure 3M). According to the subtype of cold-hot tumor, CuproCluster B with a low level of immune infiltration can be defined a “cold tumor” and CuproCluster A with a high level of immune infiltration could be considered a “hot tumor.” Furthermore, there was a significant difference in the distribution of immune subtypes between CuproCluster A and CuproCluster B (Figure 3N), and the immune checkpoint genes were significantly downregulated in CuproCluster B (Figure 3O).

### Gene clusters and characteristics

**FIGURE 4:**
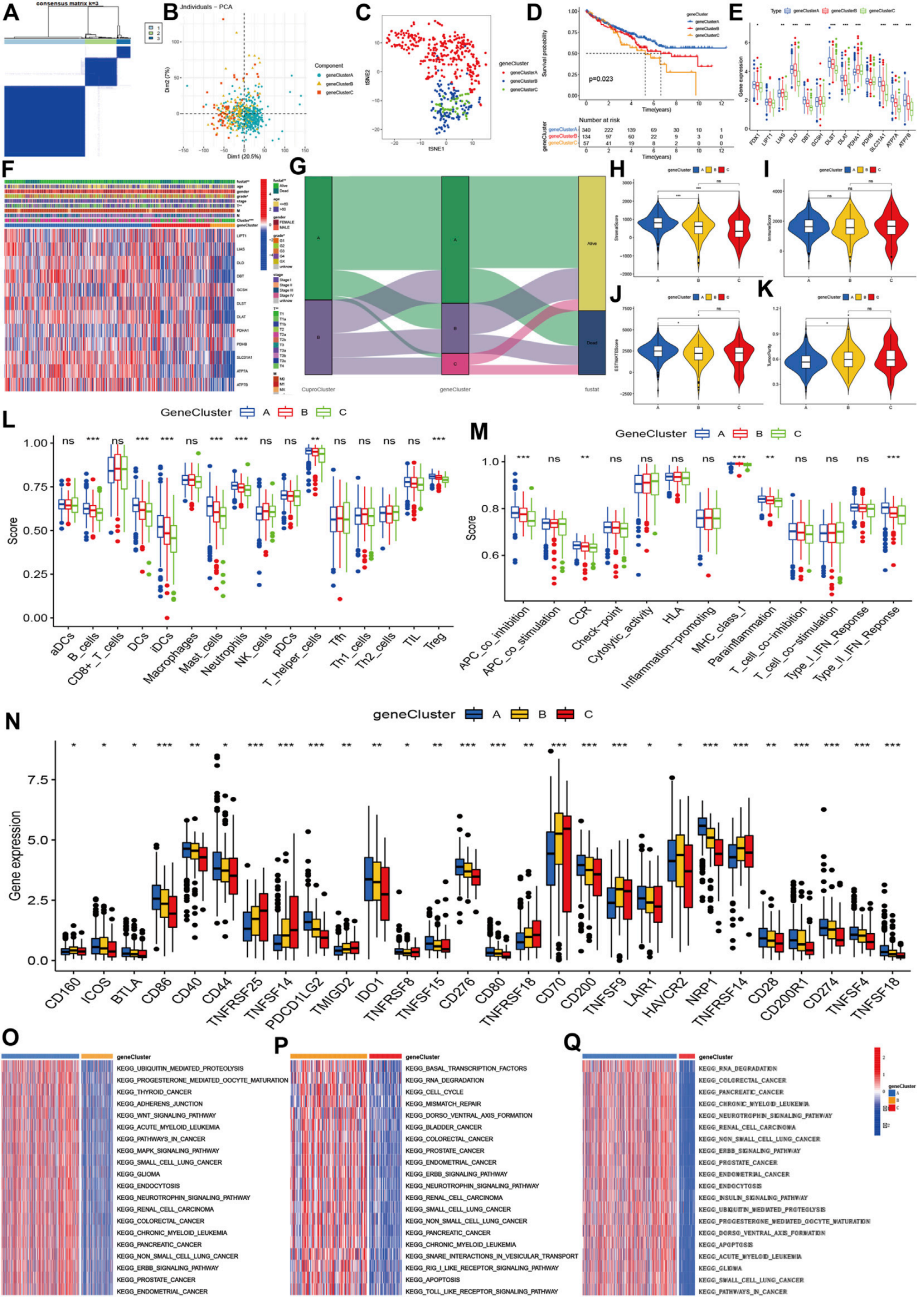
Molecular clustering based on differentially expressed genes between two cuproptosis subtypes. **(A)**: The consensus matrix identified the optimal gene clusters. **(B,C)**: PCA and tSEN identified three gene clusters. **(D)**: Kaplan-Meier survival curves of gene clusters. **(E,F)**: Correlations of gene clusters with clinical characteristics and expression of cuproptosis regulators. **(G)**: Association between cuproptosis clusters, gene clusters, and prognosis. **(H–K)**: stromal and immune estimate scores, and tumor purity across three gene clusters. **(L,M)**: Immune cells and levels of function of three gene clusters. **(N)**: Expression of immune-related checkpoint genes among three groups. **(O–Q)**: Differentially expressed signaling pathways among three gene clusters.

Using the 6742 DGEs between CuproCluster A and CuproCluster B of cuproptosis (Supplementary Table S4), we first performed clustering to identify the three gene groups (geneCluster A, geneCluster B, and geneCluster C, Figure 4A and Supplementary Table S5). Similarly, PCA and tSEN also identified three components (Figures 4B,C). Furthermore, Kaplan-Meier analysis indicated that gene Cluster C had the poorest prognosis followed by gene Cluster B, while gene Cluster A had the best OS (Figure 4D). Cuproptosis regulators also presented the highest levels of expression in gene geneCluster A among three gene clusters (Figure 4E). Next, we explored the correlations of geneClusters with clinical characteristics and cuproptosis clusters, and the results indicated that geneClusters were significantly correlated with grade, T classification, CuproClusters and clinical outcomes (Figures 4F,G). The analysis of the TME revealed that the stromal and ESTMATE scores of geneCluster A were higher than those of geneCluster B and C, while there were no differences in immune score between three geneClusters (Figures 4H–K). Furthermore, B cells showed the highest infiltration level in geneCluster A followed by geneCluster B and geneCluster C. Immune cells (DCs, iDCs, mast cells, T-helper cells, and Treg) and immune function (APC co-inhibition, CCR, class I major histocompatibility complex, pro-inflammatory, Type II interferon response) showed similar trends (Figures 4L,M). Further analyzes indicated that PD-L1, CD40, CD44, CD80, CD28, CD200, and CD86 expression decreased from gene cluster A, B, to C (Figure 4N). GSVA revealed that geneCluster A was mainly enriched in the Wnt signaling pathway, mitogen-activated protein kinase signaling pathway, neurotrophic signaling pathway, ERBB signaling pathway (Figure 4O). GeneCluster B was enriched in basal transcription factors, RNA degradation, cell cycle, the RIG I receptor signaling pathway, and receptor toll signaling pathways (Figure 4P). GeneCluster C was enriched in endocytosis, the insulin signaling pathway, and progesterone-mediated oocyte maturation (Figure 4Q and Supplementary Tables S6–S8).

### Development and validation of a cuproptosis prognostic model

**FIGURE 5:**
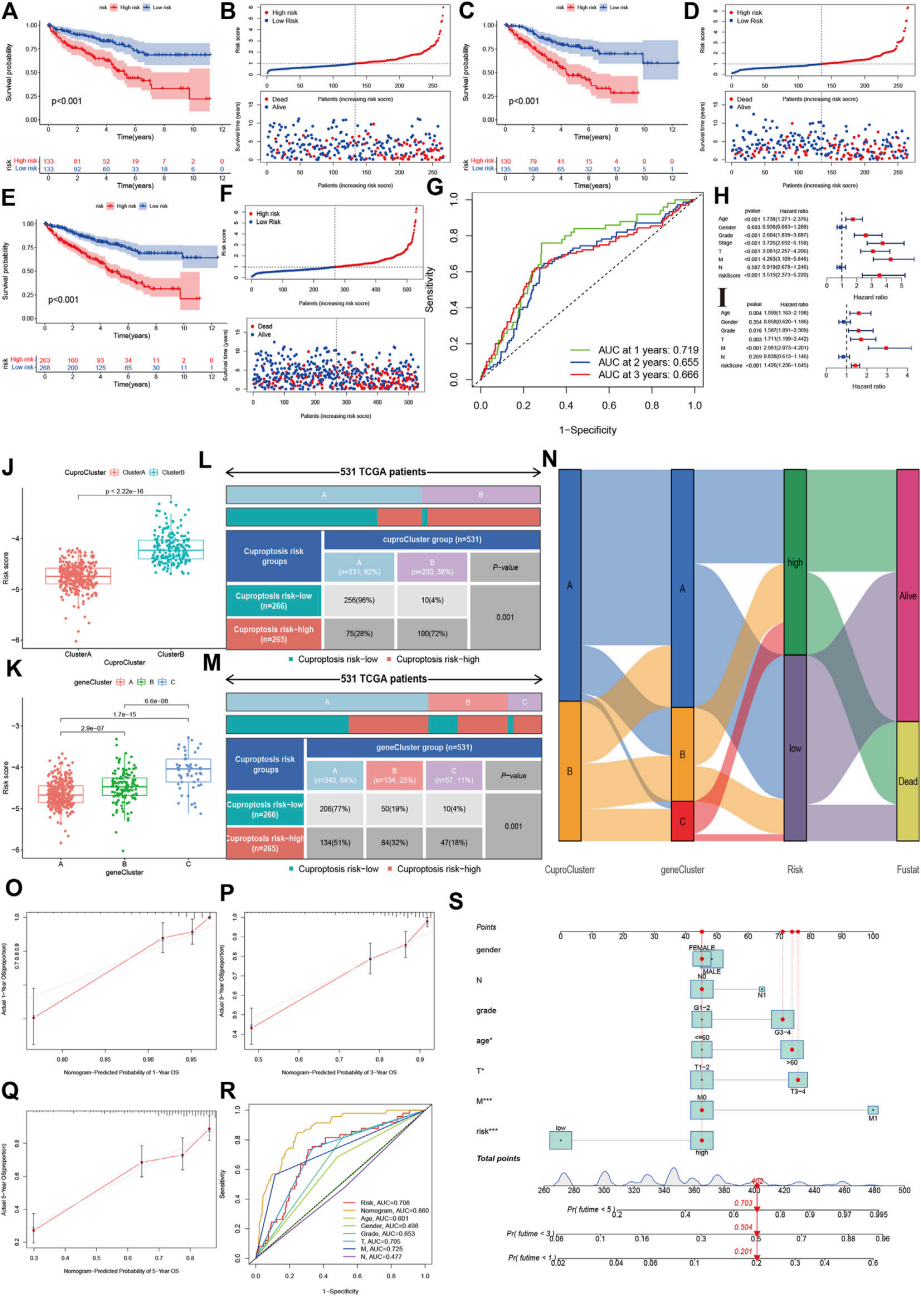
Development and validation of the cuproptosis-related prognostic model. **(A,B)**: The Kaplan-Meier (K–M) survival curve and risk distribution of the patients in the training group. **(C,D)**: The K-M survival curve and the risk distribution of the patients in the validation group. **(E,F)**: The K-M survival curve and risk distribution of patients in the whole dataset. **(G)**: The time-independent ROC of 1-year, 2-year, and 3-year overall survival. **(H,I)**: Forest plot of univariate and multivariate Cox regression for the risk score. **(J,K)**: Risk scores for cuproptosis clusters and gene clusters. **(L–N)**: Correlations of risk groups with cuproptosis clusters, gene clusters, and clinical outcomes. **(O)**: Nomogram that predicts individual risk based on risk groups and clinical parameters. **(P–S)**: The calibration plot of the observed value and the probability predicted by the nomogram at 1 year, 3 years, and 5 years. **(R)**: AUCs of the risk score and clinical parameters.

Using a training dataset, we performed Lasso regression and identified two genes in the final regression model (Supplementary Figures S1A,B). We estimated the risk score of each sample in the training group, and divided KIRC into high- and low-risk groups. Kaplan-Meier analysis showed that the high-risk group had poorer OS survival than the low-risk group (Figures 5A,B). Similar results were also found in the validation group (Figures 5C,D). The combined data also indicated that patients with a high-risk score had a poor prognosis (Figures 5E,F). Time-independent ROC showed that the AUCs for 1-year, 2-year and 3-year were 0.719, 0.655, and 0.666, respectively (Figure 5G).

### Independence and clinical correlation analysis

The univariate cox regression indicated that the risk score was associated with a poor prognosis in KIRC (hazard ratio [HR]: 3.519, 95% confidence interval [CI]: 2.373–5.220, *p* < 0.001), and the multivariate cox regression further showed that the risk score was an independent predictive factor for KIRC (HR:1.426, 95%CI:1.236–1.645, *p* < 0.001). The risk score in CuproCluster B was significantly higher than in CuproCluster A (*p* < 0.001, Figure 5J). The risk scores also increased in gene clusters A, B, and C (Figure 5K). The correlation analysis indicated that the high-risk group had a higher ratio of patients with CuproCluster B and geneClusters B and C (Figures 5L–N).

We then built a nomogram to predict the probability of risk of individual patients with KIRC (Figure 5O). The results indicated that the 1-year, 3-year and 5-year death risk were 0.201, 0.504, and 0.703, respectively, for a female aged >60 years with N1, stages III-IV, grades 3–4, and high-risk status. The 1-year, 3-year and 5-year OS calibration plots showed that the probability predicted by the nomogram was fitted with the actual observed probability (Figures 5P–R). The time-independent ROC indicated that the nomogram achieved the highest predictive value for 5-year survival outcomes, and the AUC was 0.860 (Figure 5S).

### Gene alterations and methylation

**FIGURE 6:**
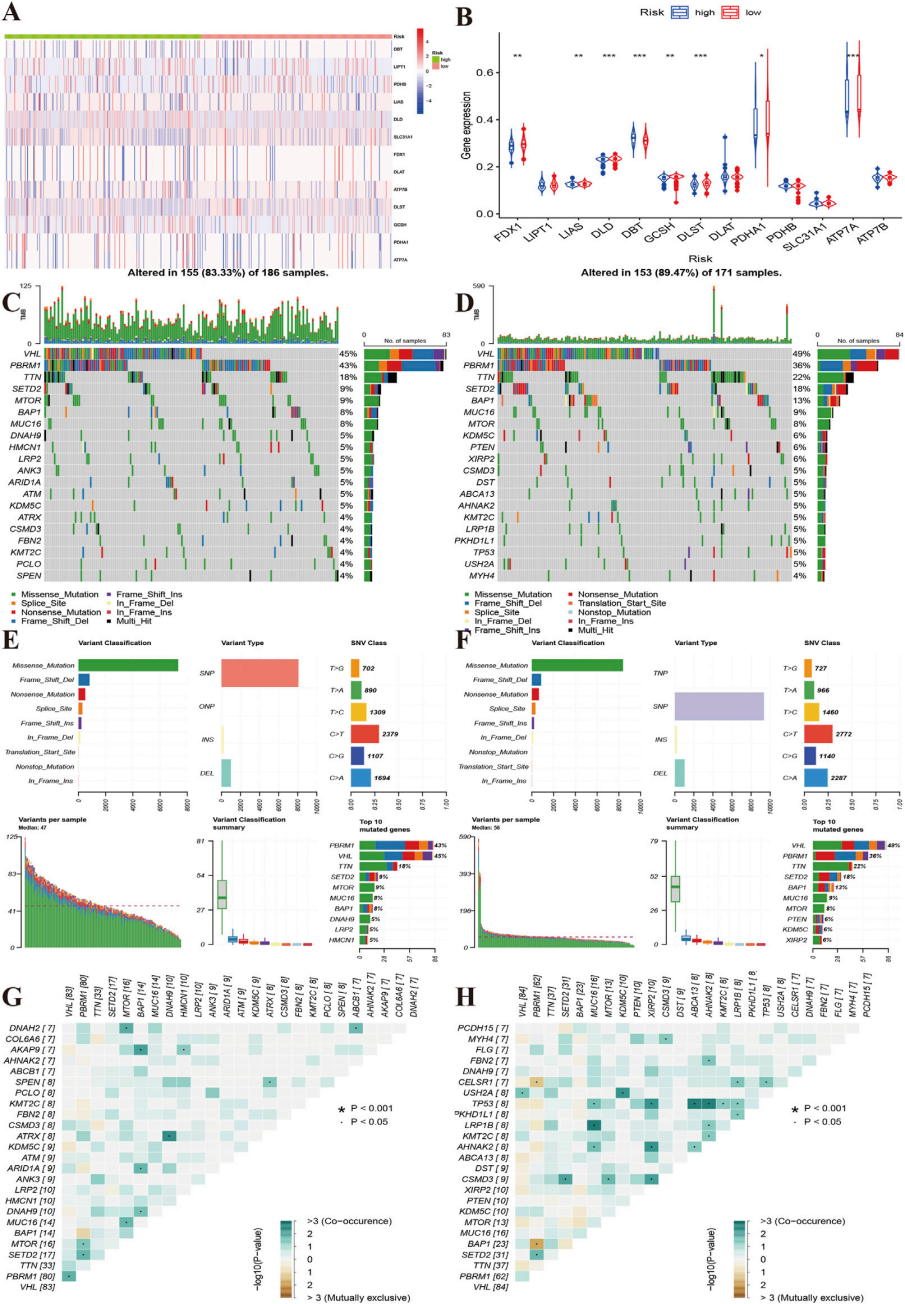
Characteristics of gene mutations of different risk groups. **(A)**: Heat map showing the alterations of the cuproptosis regulator genes between the high and low-risk groups. **(B)**: The yellow line indicates the level of methylation of the cuproptosis regulators between the high- and low-risk groups. **(C,D)**: Top 20 gene alterations of the high and low risk groups. **(E,F)**: Types of gene alterations in the high- and low-risk groups. **(G,H)**: Summary of gene co-occurrences and mutually exclusive genes of the two risk groups.

Additionally, we compared gene alterations and methylation level. We found that the high-risk group had relatively more frequent gene alterations in ATP7B and DLD (Figure 6A). FDX1, LIAS, DLD, GCSH, DLST, PDHA1, ATP7A methylation levels were lower in the high-risk group than in the low-risk group, and the opposite result was observed for DBT (Figure 6B). We further compared the top 20 gene mutations of two risk groups, and found that there were differences between the high and low-risk groups in SETD2, DNAH9, HMCN1, LRP2, ANK3, FBN2, which showed the highest mutation frequencies in high-risk group, while the low-risk group showed high frequencies in KDM5C, PTEN, XIRP2, DST, ABCA13, LRP1B and USH2A (Figures 6C,D). However, there were no differences in variant classification and variant types between two risk groups (Figures 6E,F). The high-risk group presented co-occurrence in DNAH2-MTOR, BAP1-AKAP9, DNAH9-ATRX, while the low-risk group showed mutual exclusiveness in AHNAK2/KMT2C/MUC16/XIRP2/ABSCA13/LRP1B-TP53 (Figures 6G,H).

### Correlations of the risk score with function and pathway enrichment and immune infiltration

Furthermore, we evaluated the correlations of the risk score with immune cell levels using Spearman’s analysis. The risk score was positively associated with memory B cells, regulatory T cells, follicular helper T cells, plasma cells, macrophages M0, and RNA (Supplementary Figure S2B). However, the risk score showed negative associations with eosinophils, dendritic cells, monocytes, resting mast cells, and M2 and M1 macrophages (Supplementary Figure S3). These results indicated that the high-risk group had a lower level of immune infiltration.

### Chemotherapy sensitivity and immunotherapy

**FIGURE 7:**
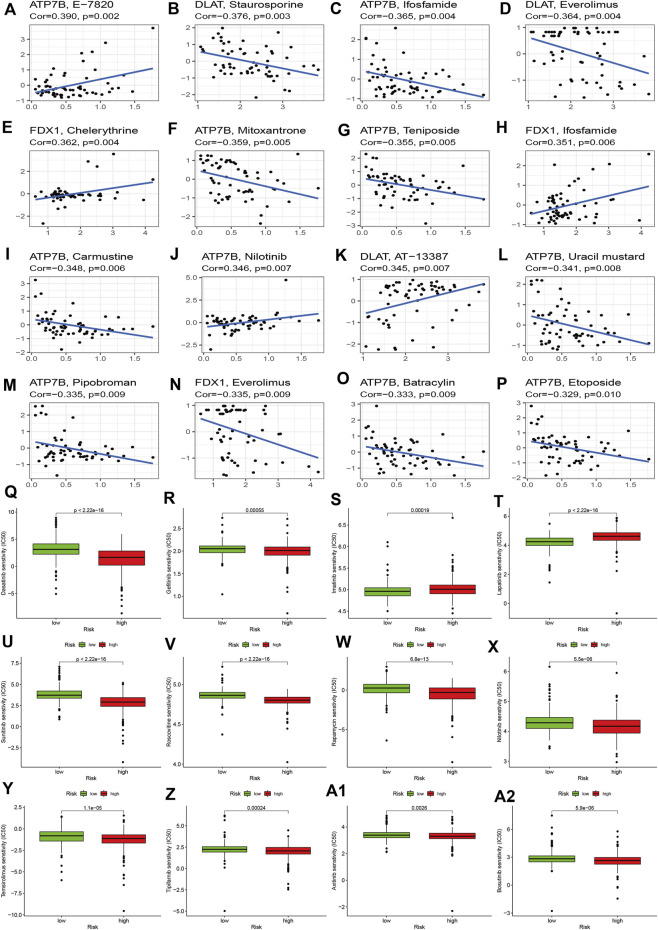
Effects of cuproptosis regulators on chemotherapy sensitivity. **(A–P)**: Small molecular compounds related to cuproptosis regulators. **(Q–A2)**: IC50 levels of different chemotherapy drugs between the high- and low-risk groups.

To explore the effects of cuproptosis regulators on treatment in KIRC, we first explored the correlations of signature genes with small molecular compounds and find ATP7B could increase resistance to chemotherapy of E-7820, carmustine, and nilotinib and showed sensitivity to chemotherapy towards ifosfamide, teniposide, mitosantrone, carmustine, and uracil mustard. DLAT expression could increase the KIRC sensitivity toward staurosporine, everolimus, and AT-13387. FDX1 expression show chemotherapy resistance of KIRC toward chelerythrine and ifosfamide (Figures 7A–L). We then compared the IC50 levels of some chemotherapy drugs between the high and low-risk groups. The results indicated that the high-risk group may show sensitivity to chemotherapy in dasatinib and gefitinib (Figure 7Q–7R). Most drug resistance was associated with the high-risk group (Figures 7S–A2).

**FIGURE 8:**
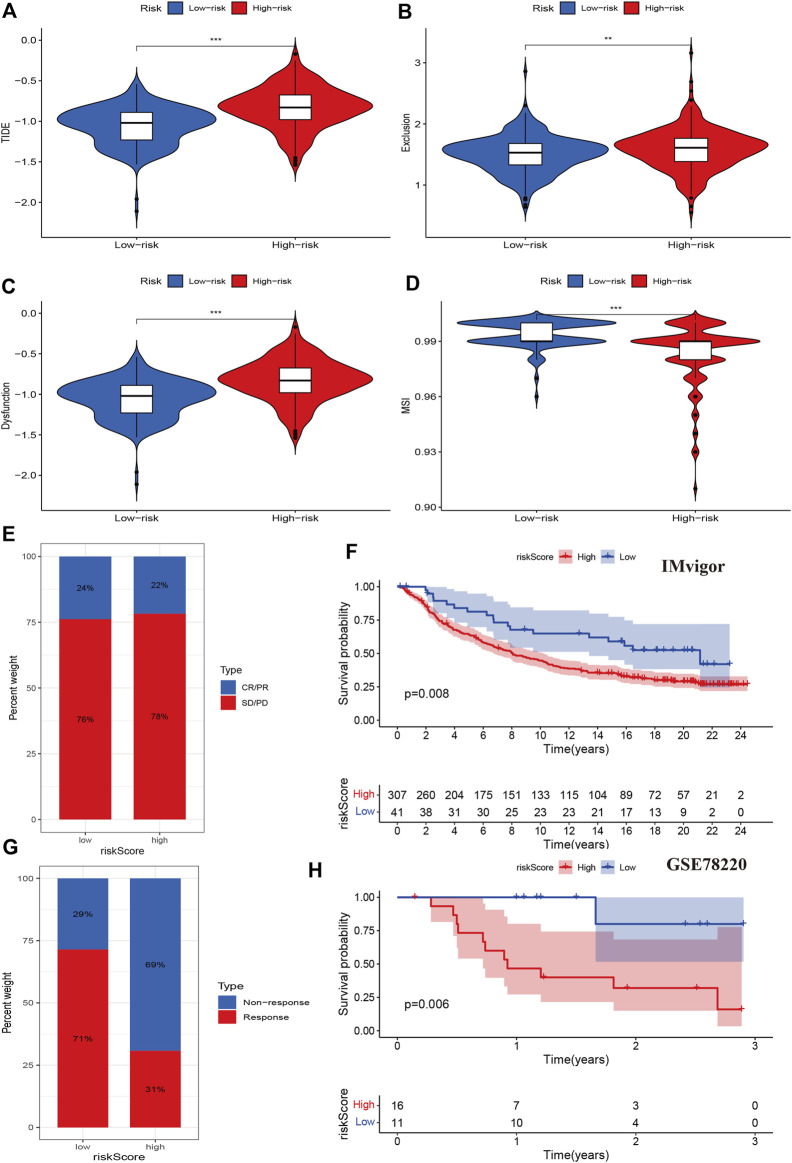
Associations between cuproptosis regulators and immunotherapy. **(A–D)**: TIDE, exclusion, dysfunction, and MSI levels of different risk groups. **(E,F)**: The immune response and prognosis comparisons of different risk groups in the IMvigor cohort. **(G,H)**: The immune response and prognosis comparisons of different risk groups in the GSE78220 cohort.

We then evaluated tumor immune dysfunction and exclusion (TIDE). Based on tumor pretreatment expression profiles, this TIDE module can estimate multiple published transcriptomic biomarkers to predict patient response. The results indicated that the high-risk group had a higher level of TIDE, exclusion, and dysfunction than the low-risk group, while the degree of microsatellite instability (MSI) of the high-risk group decreased significantly (Figures 8A–D). Finally, we evaluated the effects of the cuproptosis risk score in patients who received PD-L1 inhibitors. The CR/PR response rate was similar between the high- and low-risk groups (78% vs. 76%, *p* > 0.05, Figure 8E), but the high-risk group had a poorer prognosis than the low-risk group (*p* = 0.008, Figure 8F). In other data from the immunotherapy cohort, the high-risk group had a lower immunotherapy response rate than the low-risk group (31% vs. 71%, *p* < 0.001), and the Kaplan-Meier analysis indicated that the prognosis of the high-risk group was poorer than that of the low-risk group (*p* = 0.006, Figure 8H). These results indicated that cuproptosis regulators could affect the immunotherapy response and efficiency in cancer.

## Discussion

Copper ion is an essential metal element in living organisms. It plays an important role in biological mechanisms acting as a cofactor of essential enzymes (Mahl et al., 2020). In the normal physiological state, copper ions maintain low concentration and dynamic balance in the organism, when abnormal accumulation of copper ions can cause copper toxicity and then induce cell death (Fowler et al., 2019). Studies have shown that mutations in human genes can cause an imbalance of copper homeostasis and induce a variety of diseases (Wang et al., 2022a; Tang et al., 2022). Furthermore, several studies have found that copper ion carriers and their chelating agents are expected to be potential drug molecules for tumor treatment (Cobine and Brady, 2022). However, the specific molecular mechanisms underlying copper ion-induced cell death have not been clarified. Therefore, exploring mechanisms of copper-induced cell death is helpful to better understand the copper imbalance and to develop treatment strategies.

Recent studies also evaluated the role of cuproptosis in KIRC (Bian et al., 2022; Ji et al., 2022; Wang and Wang, 2022). However, there were notable differences with the present study. First, we performed a molecular cluster analysis and identified two cuproptosis clusters and three gene clusters that could facilitate personalized treatment, which were not reported in the Bian’s and Wang’s study but three cuproptosis clusters in Li’s study. Second, we build a prognostic model using different processing strategies. We divided the whole sample into training and test cohorts. Then, we built the prognostic model in the training cohort and validated the prognostic model in the test cohort. The previous studies only built prognostic models that were not validated using external or even internal data. Besides, the aforementioned model for OS included multiple genes (Bian: FDX1, DLAT, CDKN2A; Wang: MTF1, LIAS, FDX1, DLAT, CDKN2A; Li: ENAM, WDR72, CLDN10, HMGCS2, CYFIP2, and QRFPR) with low predictive ability, while our model included two genes (FDX1 and DBT) with 0.714, 0.660, and 0.684 at the 1-year, 3-year, and 5-year time points. Our model showed a high predictive capacity. Third, we included key genes as markers to build the nomogram, which differed from the previous study that used single genes in the final model, while Wang and Li’s studies did not perform such analyses. Our final nomogram risk score achieved 0.860 AUC at the 5-year time point. Furthermore, we evaluated the sensitivity to chemotherapy of the model and its effects on immunotherapy in data from two independent cohort, which was also not reported in the previous three studies. Finally, we evaluate the effect of cuproptosis on immunotherapy in two independent cohort data, which were not reported in previous three studies. Overall, our study provided more comprehensive results, an efficient and reliable prognostic model, and a strategy for practical clinical treatment.

Precision oncology requires that clinical practitioners use the molecular characteristics of individual patient tumors to assess the benefit or toxicity of specific therapeutic interventions (Mateo et al., 2022). If we can use genomics to classify tumors into molecular subtypes with oncogenic mechanisms and responses to targeted drugs against these mechanisms, this will have important implications for our understanding of the molecular heterogeneity that is prevalent between different tumor subtypes and can promote the development of clinical therapeutic drugs (Prasad et al., 2016). Our results indicate that patients with KIRC can be divided into two molecular subtypes with different biological functions, clinical prognosis, and immune status according to the expression of cuproptosis regulators. Our findings may be helpful for the management of the risk of KIRC. Furthermore, we obtained three clusters of genes from the DGE analysis between two molecular subtypes of cuproptosis, which will help stratify intervention management with greater precision. Next, we built a cuproptosis prognostic model that could predict the OS of KIRC. Compared to the previous prognostic model comprising multiple and even more than a dozen genes, our model was more practical as our prognostic model only included two genes: FDX1 and DBT. FDX1 encodes a small iron-sulfur protein that transfers electrons from NADPH through ferredoxin reductase to mitochondrial cytochrome P450, and is involved in the metabolism of steroids, vitamin D, and bile acids (Mateo et al., 2022). DBT encodes an inner mitochondrial enzyme complex involved in the breakdown of branched chain amino acids isoleucine, leucine, and valine (Li et al., 2021; Wang et al., 2022b). A recent study found that copper-dependent cell death occurs through the direct binding of copper ions to the lipoacylated components of the tricarboxylic acid cycle (TCA) in mitochondrial respiration, resulting in the aggregation of lipoacylated proteins and subsequent down-regulation of iron-sulfur clusters, which leads to proteotoxic stress and ultimately to cell death. FDX1 and DBT are important cuproptosis regulators that can induce cell death through copper ions, and may be a new method of tumor therapy (Tsvetkov et al., 2022). Our results indicated that high expression of cuproptosis-inducing FDX1 and DBT was significantly associated with a favorable prognosis in KIRC. Therefore, increasing the expression of FDX1 and DBT could be a potential approach for killing tumor cells in KIRC. This may be potential mechanism of cuproptosis inducing cell death in KIRC.

Subsequently, we established a nomogram risk score system, and calibration analyses and time-independent ROC proved that this individual risk prognosis model achieved good prediction ability. Furthermore, we divided KIRC patients into high- and low-risk groups according to the risk score. Different risk groups presented different biological functions and signaling pathways. GO enrichment analysis indicated that the high-risk group was enriched in the protein catabolic process, neutrophil activation and degranulation, immune response, protein ligase, GTPase binding, and KEGG pathway analysis revealed that cytokine-cytokine receptor interaction, the IL-17 signaling pathway, epithelial cell signaling, valine, leucine, and isoleucine degradation were highly enriched in the high-risk group. These functions and pathways are suggested to be associated with KIRC (Akhtar et al., 2018).

The TME and immune infiltration play an important role in tumor genesis and development (Pages et al., 2010; Fridman et al., 2011; Wu and Dai, 2017; Arneth, 2019). Our results also indicated that the risk score was positively associated with memory B cell, regulatory T cells, follicular helper T cells, plasma cells, M0 macrophages and showed negative associations with eosinophils, dendritic cells, monocytes, mast cells resting, and M2 and M1 macrophages. These results indicated that the high-risk group had lower levels of immune infiltration. A positive response to immunotherapy usually depends on the interaction between tumor cells and immune regulation within the TME. In these interactions, the TME plays an important role in inhibiting or enhancing the immune response (Kim et al., 2020; Mhaidly and Mechta-Grigoriou, 2021). Therefore, we evaluated the effect of cuproptosis regulators on immunotherapy. We first examined tumor immune dysfunction and exclusion and found that the high-risk group had a higher level of TIDE, exclusion and dysfunction than the low-risk group, while the MSI level of the high-risk group significantly decreased, which means that the high-risk group may have a poor response to immunotherapy. The cytotoxic effect of copper carrier is related to mitochondrial respiration. Previous studies have found that when the mitochondria of innate immune cells are divided, melanoma growth is significantly reduced and survival of vaccinated animals is significantly improved. Tumor-infiltrating T cells also produce large amounts of IFN-γ, which enhances their anti-tumor immunity (Gao et al., 2017). In addition, the relationship between copper death and immune regulation may be related to NK cell metabolism. Activated NK cells not only have enhanced glycolysis supported by increased expression of glycolytic enzymes and glucose transporters, but also have increased basal oxidative phosphorylation rate and maximal respiratory capacity accompanied by increased mitochondrial mass. The insufficient metabolism of NK cells in tumor microenvironment will weaken their immune surveillance of tumor, and is related to tumor growth and metastasis (Guo et al., 2022). This may be one of the mechanisms by which copper death affects KIRC immune regulation.

To validate this finding, we evaluated the effect of the cuproptosis risk score on patients who received PD-L1 inhibitors. The IMvigor tool showed that the response rate was similar between the high- and low-risk groups, but the high-risk group had a poorer prognosis than the low-risk group. In another immunotherapy cohort data, the high-risk group had lower immunotherapy response rate and poorer prognosis than low-risk group. Our findings confirmed the immune regulation function of cuproptosis in KIRC. Finally, we identified several small molecular compounds that are resistant and sensitive to chemotherapy, which can help clinicians make decisions in clinical practice.

The present study presented several limitations that should be considered. One limitation was that the validation data consisted of internal data and additional data are required to validate the prognostic model and immunotherapy response. Another limitation was that our study did not define a biological mechanism. We can only explore the molecular mechanism through omics data, and *in vitro* and *in vivo* experiments should be performed to confirm the present findings. However, these multiple omics analyses based on cuproptosis regulators will help in stratifying risk management and in providing a better understanding of the molecular mechanisms and treatment strategies for KIRC.

In conclusion, cuproptosis regulators can be used for molecular subtyping and prognostic risk assessment in KIRC. The established nomogram risk score system presented a high predictive capacity for the prognosis of individual patients. Furthermore, cuproptosis regulators can influence the response to chemotherapy and immunotherapy in KIRC. The present study provides new information and theoretical support that will facilitate risk management and individualized treatment in KIRC.

## Data Availability

Publicly available datasets were analyzed in this study. The names of the repository/repositories and accession number(s) can be found in the article/Supplementary Material.
