# Supplementary material for: Molecular subtypes based on cuproptosis regulators and immune infiltration in kidney renal clear cell carcinoma
Source: Front Genet. 2022 Oct 21;13:983445. doi: 10.3389/fgene.2022.983445 (PMC9635053; doi:10.3389/fgene.2022.983445)
Supplement: Supplementary file 2 [file Table1.DOCX]

**Supplementary material legends**

**Figure S1 A and B:** LASSO regression identified the optimal number of genes in the model. **C and D:** Time-independent ROC evaluated the predictive ability of risk score in training and validating dataset.

**Figure S2 A:** GO enrichment analysis for DGEs between high- and low- risk group. **B:** KEGG pathway analysis between high- and low- risk group.

**Figure S3** Correlations of risk score with immune cells.

**Table S1** The 13 cuproptosis-associated genes used for classification

**Table S2** KIRC classification pattern based on cuproptosis-related genes

**Table S3** Differentially expressed genes between Cluster A and Cluster B based on cuproptosis-related genes

**Table S4** The geneCluster based on cuproptosis-correlated differentially expressed genes

**Table S5** GSVA enrichment between gene cluster A and gene cluster B

**Table S6** GSVA enrichment between gene cluster B and gene cluster C

**Table S7** GSVA enrichment between gene cluster A and gene cluster C
