# Supplementary figures and images for: Molecular subtypes based on cuproptosis regulators and immune infiltration in kidney renal clear cell carcinoma
Source: Front Genet. 2022 Oct 21;13:983445. doi: 10.3389/fgene.2022.983445 (PMC9635053; doi:10.3389/fgene.2022.983445)

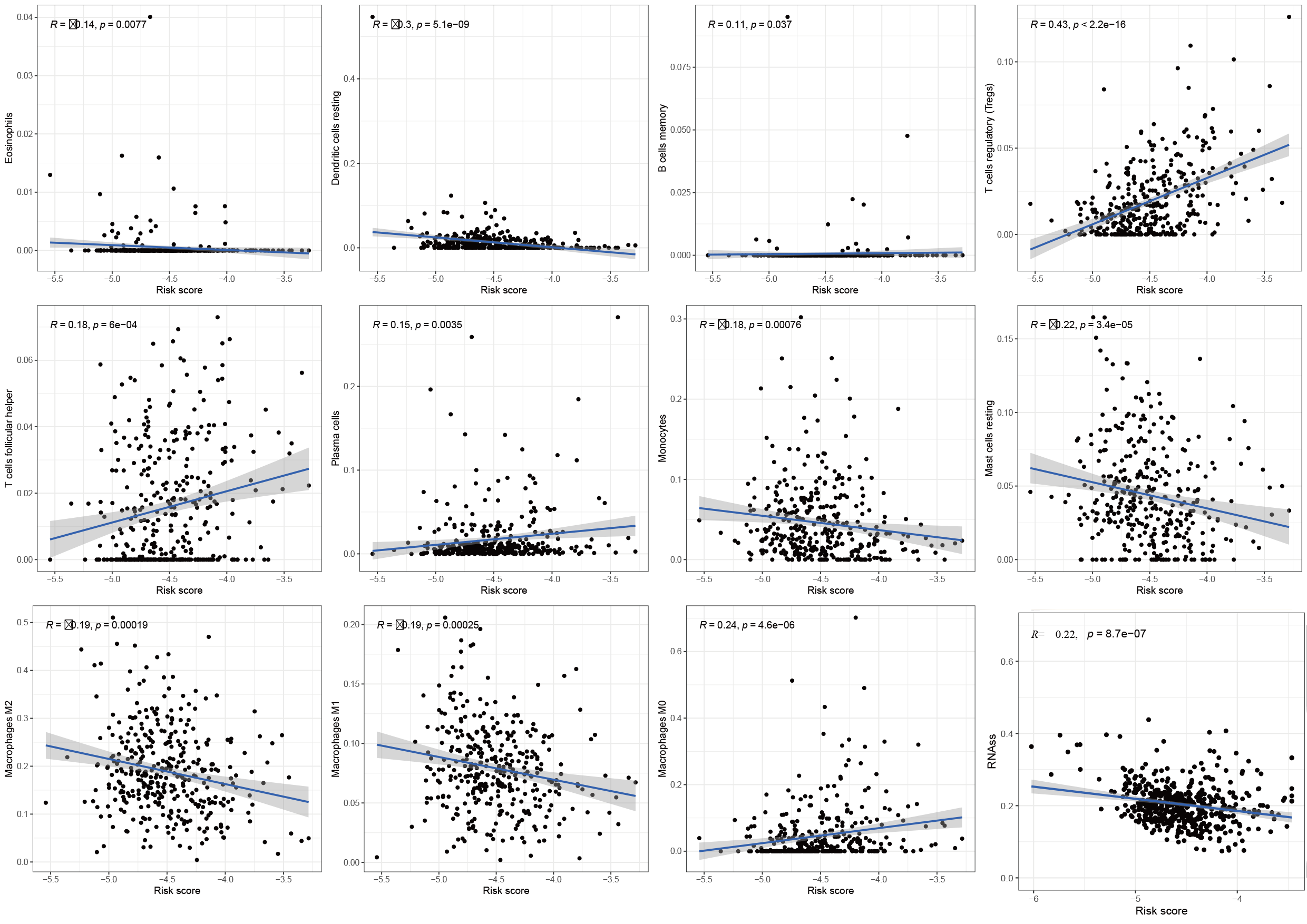

Supplement: Supplementary file 3 [file Image3.TIF]

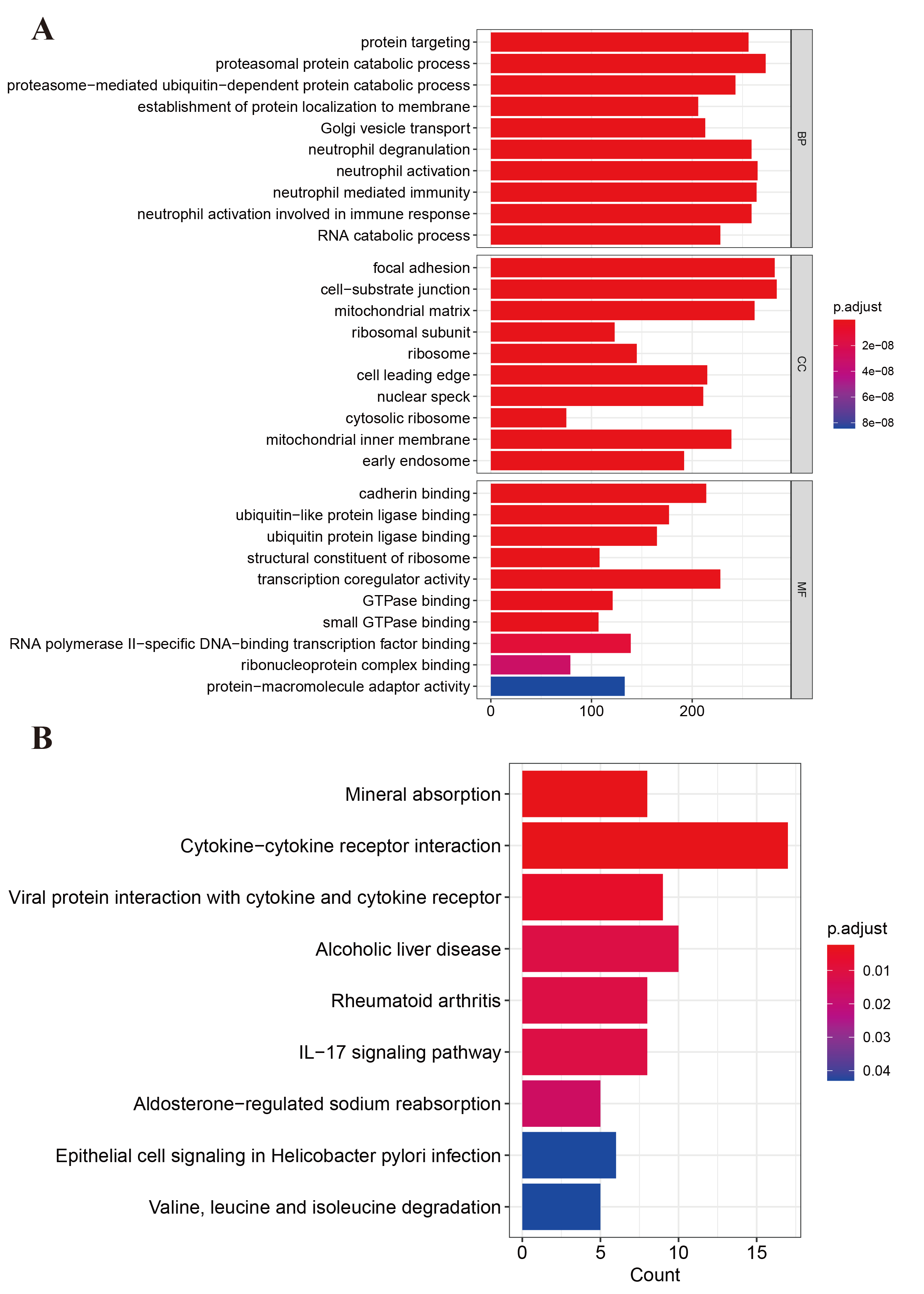

Supplement: Supplementary file 4 [file Image2.TIF]

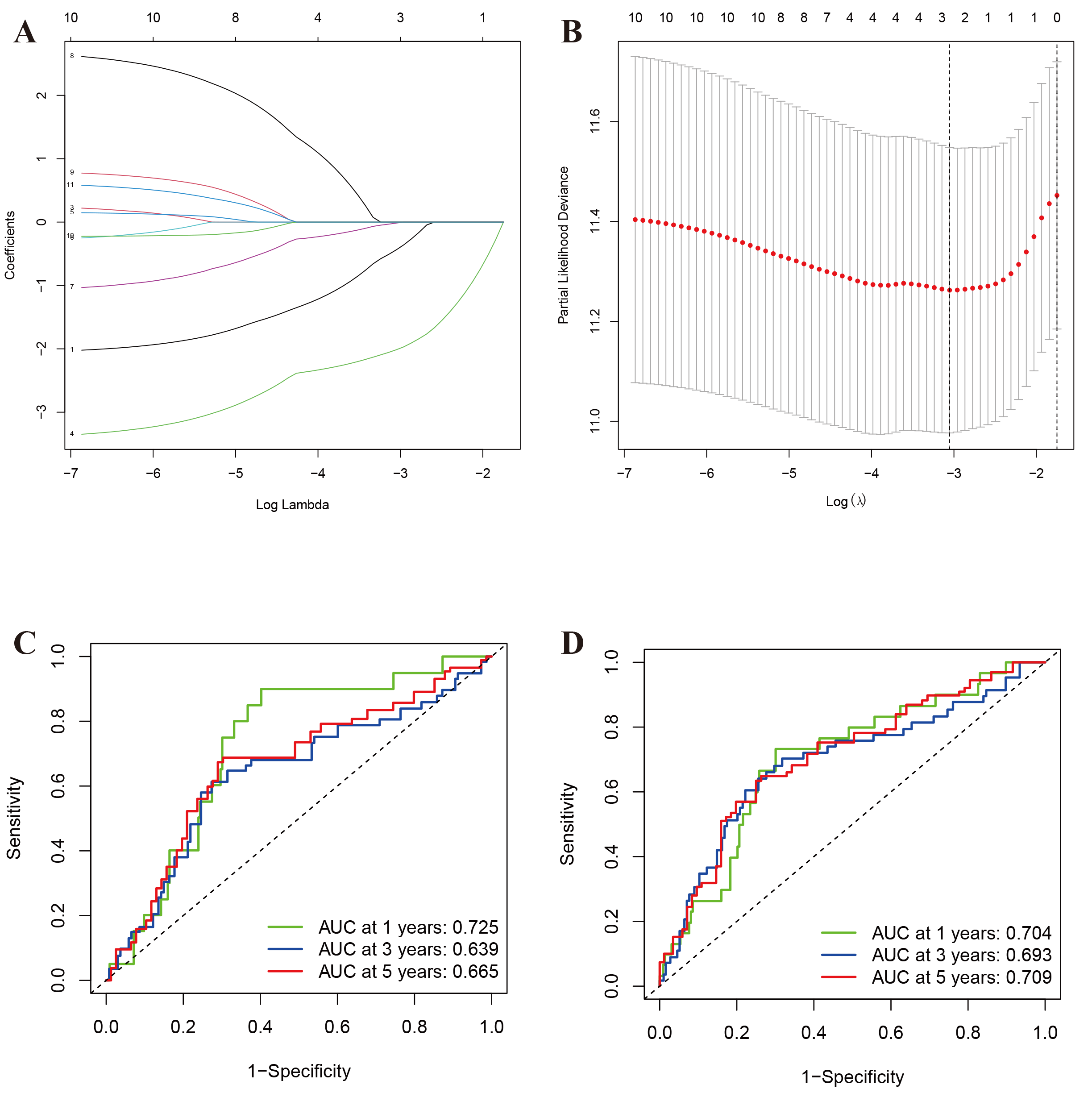

Supplement: Supplementary file 5 [file Image1.TIF]
